# Polygenic hazard score modified the relationship between hippocampal subfield atrophy and episodic memory in older adults

**DOI:** 10.3389/fnagi.2022.943702

**Published:** 2022-10-31

**Authors:** Jingjing Xu, Xiaojun Guan, Jiaqi Wen, Minming Zhang, Xiaojun Xu, for the Alzheimer’s Disease Neuroimaging Initiative

**Affiliations:** Department of Radiology, The Second Affiliated Hospital, Zhejiang University School of Medicine, Hangzhou, China

**Keywords:** Alzheimer’s disease, hippocampus subfield, episodic memory, polygenic hazard score, volume ratio

## Abstract

**Background:**

Understanding genetic influences on Alzheimer’s disease (AD) may improve early identification. Polygenic hazard score (PHS) is associated with the age of AD onset and cognitive decline. It interacts with other risk factors, but the nature of such combined effects remains poorly understood.

**Materials and methods:**

We examined the effect of genetic risk and hippocampal atrophy pattern on episodic memory in a sample of older adults ranging from cognitively normal to those diagnosed with AD using structural MRI. Participants included 51 memory unimpaired normal control (NC), 69 mild cognitive impairment (MCI), and 43 AD adults enrolled in the Alzheimer’s Disease Neuroimaging Initiative (ADNI). Hierarchical linear regression analyses examined the main and interaction effects of hippocampal subfield volumes and PHS, indicating genetic risk for AD, on a validated episodic memory composite score. Diagnosis-stratified models further assessed the role of PHS.

**Results:**

Polygenic hazard score moderated the relationship between right fimbria/hippocampus volume ratio and episodic memory, such that patients with high PHS and lower volume ratio had lower episodic memory composite scores [ΔF = 6.730, *p* = 0.011, Δ*R*^2^ = 0.059]. This effect was also found among individuals with MCI [ΔF = 4.519, *p* = 0.038, Δ*R*^2^ = 0.050]. In contrast, no interaction effects were present for those NC or AD individuals. A follow-up mediation analysis also indicated that the right fimbria/hippocampus volume ratio might mediate the link between PHS and episodic memory performance in the MCI group, whereas no mediation effects were present for those NC or AD individuals.

**Conclusion:**

These findings suggest that the interaction between AD genetic risk and hippocampal subfield volume ratio increases memory impairment among older adults. Also, the results highlighted a potential pathway in which genetic risk affects memory by degrading hippocampal subfield volume ratio in cognitive decline subjects.

## Introduction

Alzheimer’s disease (AD) is the most frequent cause of dementia (Prince et al., 2013). Clinically, AD develops gradually and presents with progressive decline in multiple cognitive domains, particularly affecting episodic memory (Small et al., 1997; Elias et al., 2000). As the Chinese population is aging, more than seven million Chinese people live with AD currently, and the costs are predicted to reach US $507.49 billion in 2030 (Jia et al., 2018). It is, therefore, important to gain a better understanding of the factors associated with dementia, including brain and genetic markers. Understanding how genetic risk for AD affects the brain might shed light on mechanisms leading to AD cognitive decline later in life.

Studies have shown that genetic risk factors play a critical role in AD development (Gatz et al., 2006). Various susceptibility loci have been identified. For example, early-onset AD is considered to be caused by mutations in the amyloid precursor protein (APP), presenilin-1 (PRES-1), and presenilin-2 (PRES-2) genes (Goate et al., 1991; Levy-Lahad et al., 1995; Rogaev et al., 1995). The apolipoprotein E4 allele (APOE) is the most well-known genetic risk factor linked to late-onset AD (Strittmatter et al., 1993; Gatz et al., 2006; Bettens et al., 2013). But the majority of studies have focused on the risk associated with a single-candidate gene (Strittmatter et al., 1993; Wang et al., 2019). Based on a combination of APOE and 31 other genetic variants, a polygenic hazard score (PHS) has been developed and validated for quantifying AD dementia age of onset (Desikan et al., 2017). The PHS showed substantial improvement over APOE in predicting the age of AD onset and was associated with biomarkers of AD, including MRI-based hippocampal volume loss and cognitive impairment (Desikan et al., 2017; Kauppi et al., 2018).

The genetic effects on AD cognitive decline can be revealed *in vivo* in the human brain by structural and functional magnetic resonance imaging (MRI) methods. Structural MRI studies have provided evidence of hippocampal atrophy as a key factor in memory impairment in AD (Zarow et al., 2011; Weiner et al., 2013; Harper et al., 2014) and hippocampal volume is considered an index of the degree of cognitive decline (Kilpatrick et al., 1997; Ystad et al., 2009). However, the hippocampal formation is not homogeneous but is composed of several interconnected subregions, namely hippocampal subfields that are believed to have different functions (Duvernoy, 2008; Mueller et al., 2011). Their specialization makes the hippocampal subfields differentially susceptible to AD pathogenic disruptions (Apostolova et al., 2010; Pluta et al., 2012; Zeng et al., 2021). But how the anatomical vulnerability in hippocampal subfields observed in AD is linked to memory impairment remains unknown. This might be partially explained by regional differences in the vulnerability to tau accumulation and neurofibrillary tangles (NFTs), which are core pathological markers of AD (Braak and Braak, 1990; Ball, 1997; Schonheit et al., 2004). Therefore, the volume of hippocampal subfields might be more sensitive imaging biomarkers for understanding memory in AD.

Previous studies suggest that memory impairment in AD may be mediated through APOE-induced changes in the hippocampus (Han and Bondi, 2008; Caselli et al., 2009; Tuminello and Han, 2011; Wang et al., 2019). First, episodic memory is critically dependent on the hippocampus and is impaired in AD. Second, both the decrease of hippocampal volume and memory dysfunction follow a similar genetic trajectory, e.g., both decrease in healthy older people with the possession of the ε4 allele. Finally, in AD, APOE, which asserted different effects on episodic memory, was associated with different compensatory recruitment processes in the hippocampus. A recent study revealed that PHS moderated the relationship between the medial temporal lobe (MTL) volume and episodic memory in older AD adults (Prieto et al., 2020). However, it is unknown whether PHS has similar effects on the association of hippocampal subfield volumes with episodic memory across the AD continuum. Our main goal was to examine whether the PHS modulates the relationship between hippocampal subfield volumes and episodic memory in subjects with normal control (NC), mild cognitive impairment (MCI), and AD from the Alzheimer’s Disease Neuroimaging Initiative (ADNI) dataset. We hypothesized that individuals with high polygenic risk for AD and smaller hippocampal subfield volume ratios would show reduced episodic memory performance. And we intended to investigate if hippocampal subfield volume ratios mediated the effect of PHS on memory scores.

## Materials and methods

### Participants

Data used in the preparation of this article were obtained from the ADNI database.^1^ The ADNI was launched in 2003 as a public–private partnership, led by Principal Investigator Michael W. Weiner, MD. The primary goal of ADNI has been to test whether serial magnetic resonance imaging (MRI), positron emission tomography (PET), other biological markers, and clinical and neuropsychological assessment can be combined to measure the progression of MCI and early AD.

We selected subjects from the ADNI-2 population based on study forms downloaded from the website. Inclusive and exclusive criteria can be found in detail at http://www.adni-info.org. The ADNI criteria for normal controls (NC) were: (1) a Mini-Mental State Examination (MMSE) score of at least 24; (2) a Clinical Dementia Rating (CDR) score of 0; and (3) no report of any cognition complaint. The ADNI criteria for MCI were: (1) subjective memory complaints; (2) objective memory loss defined by the Wechsler Memory Scale (WMS-R) logical memory test (Wechsler, 1987); (3) a global CDR score of 0.5; (4) an MMSE score of equal to, or higher than, 24 out of 30; and (5) general cognitive and functional performance sufficiently preserved such that a diagnosis of dementia could not be made by the site physician at the time of screening. Diagnostic criteria for AD included MMSE scores between 20 and 26 and a global CDR of 0.5 or 1.0 at baseline (Petersen et al., 2010).

Based on the above criteria, we identified 163 subjects, including 51 subjects with NC, 69 patients with MCI, and 43 patients with AD. Individuals with a non-accelerated T1 MRI screening scan, PHS, and baseline visit episodic memory were included for analyses. Written informed consent was obtained from all participants or their authorized representatives. All the participants from ADNI-2 with PHS and a non-accelerated T1 MRI screening scan using SPGR were included.

### Structural magnetic resonance imaging

Structural MRI brain scans were obtained with a standardized protocol, which is described in detail at www.loni.ucla.edu/ADNI. Sagittal 3D T1-weighted MRI sequence (TE/TI/TR = 2.98/900/2,300 ms, matrix size 256 × 256 × 176, slice thickness = 1.20 mm) was performed for each participant.

### Hippocampal subfields volume estimation

**FIGURE 1:**
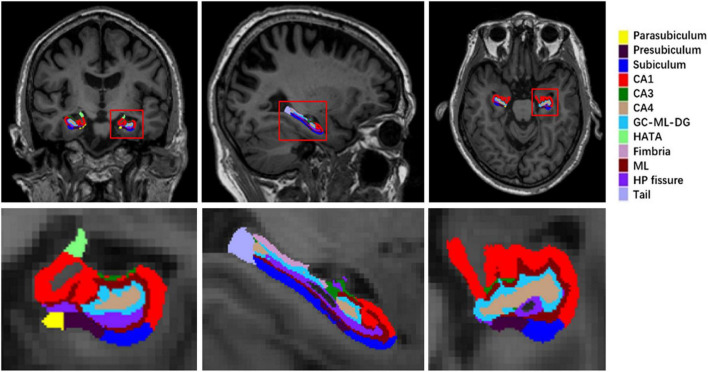
A sample of left hippocampal subfield automated segmentation. HP, hippocampus; ML, the molecular layer; CA, cornus ammonis; GC-ML-DG, the molecular and granule cell layers of the dentate gyrus; HATA, hippocampal amygdala transition area.

Volumetric measures of hippocampal subfields were performed using FreeSurfer (version 6.0).^2^ Automated segmentation of the hippocampal subfields was performed based on a computational atlas of the hippocampal formation using a combination of *ex vivo* and *in vivo* MRI data (Iglesias et al., 2015). The atlas includes the hippocampal tail, subiculum, CA1, CA3, CA4, the hippocampal fissure, presubiculum, parasubiculum, the molecular layer (ML), the molecular and granule cell layers of the dentate gyrus (GC-ML-DG), fimbria, and the hippocampal amygdala transition area (HATA). The images from a normal subject are shown in Figure 1 as an example. Also, it is important to consider the relationship of the hippocampal subfields and the entire ipsilateral hippocampus (eiHP), which varies between individuals (Burwell and Agster, 2008). As described previously, the entire hippocampal volume by FreeSurfer is an atlas-based estimation approach (Iglesias et al., 2015). In this study, we used hippocampal subfield-to-eiHP volume ratio (VR) for further statistical analyses.

### Polygenic hazard score

For all participants in this study, their individual PHS was computed, as described previously (Kauppi et al., 2018). Briefly, AD-associated single-nucleotide polymorphisms (SNPs) (at *p* < 10^–5^) were first delineated using genotype data from 17,008 AD cases and 37,154 controls from Stage 1 of the International Genomics of Alzheimer’s Project. Next, using genotype data from 6,409 patients with AD and 9,386 older controls from Phase 1 of the Alzheimer’s Disease Genetics Consortium (ADGC Phase 1), and corrected for the baseline allele frequencies using European genotypes from the 1,000 Genomes Project, a total of 31 AD-associated SNPs were identified from a stepwise Cox proportional hazards model to derive a PHS for each participant. Finally, by combining US population-based incidence rates and the genotype-derived PHS for each individual, estimates of instantaneous risk (i.e., cumulative incidence rate) for developing AD were derived based on genotype and age (Desikan et al., 2017; Tan et al., 2019). Considering APOE is strongly associated with both cognition and hippocampal volumes even in very early cases (Haller et al., 2019; Herrmann et al., 2019), we have completed all the analyses including APOE genotype as a covariate.

### Memory composite score

The composite episodic memory score was derived from the neuropsychological battery administered in ADNI (Crane et al., 2012). The memory composite score was created from the following: longitudinal Rey Auditory Verbal Learning Test (RAVLT, two versions), AD assessment schedule - cognition (ADAS-Cog, three versions), memory components of MMSE, and logical memory task. Cognitive data from 803 ADNI participants were used. Composite scores have a mean of 0 and a standard deviation (SD) of 1 (Crane et al., 2012).

### Statistical analysis

Demographics and cognitive outcomes were compared between diagnostic groups using analysis of variance (ANOVA) for continuous variables and Chi-squared for categorical variables in all subjects and within diagnostic groups.

General linear mixed models were used to evaluate the associations between diagnosis and PHS status on HP subfield volume ratios in all subjects. In this study, the model included diagnosis (normal control, MCI, and AD), PHS status (high PHS and low PHS), and the diagnosis × PHS status interaction term. Covariates were age, gender, and education. If significant subfields were identified, we calculated the difference of HP subfield VR between each diagnosis group which was found to be statistically significant at the alpha = 0.05 level in the least significant difference (LSD) test.

If significant interaction effects were observed, partial correlation analyses were conducted to determine the relationship between HP subfield VR and memory score in all subjects and within diagnostic groups regressing out the same covariates. HP subfield VR and memory scores were compared between high PHS and low PHS groups using Student’s *t*-test in all subjects and within diagnostic groups.

To verify the presence of an interaction between PHS and HP subfield VR on composite episodic memory scores, we used hierarchical linear regression models with an added interaction term. Covariates included in the first step of the linear regression model were: sex, age, and education. The second step of the model assessed for the main effects of PHS and HP subfield VR. The third step of the model added the interaction between PHS and HP subfield VR. Diagnosis-stratified analyses were conducted to establish if the effects were more prominent in a particular diagnostic group (NC, MCI, AD). The resultant *p*-values for the associations of the PHS × HP subfield VR interaction with memory performance were corrected for multiple comparisons with false discovery rate (FDR) (Benjamini and Hochberg, 1995). IBM SPSS version 19.0 was used to perform all statistical analyses. A two-tailed *p*-value of less than 0.05 was considered to be statistically significant (corrected for multiple comparisons with Bonferroni).

**FIGURE 2:**
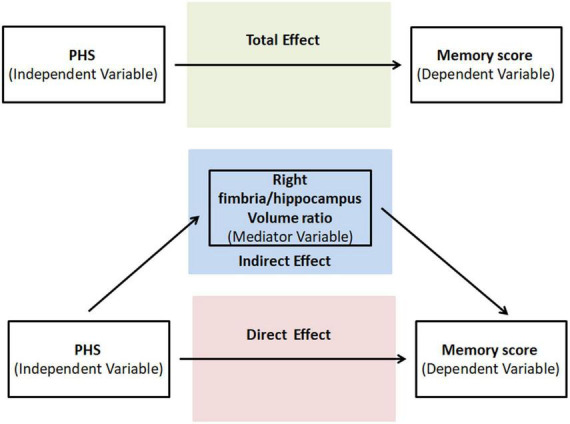
Graphical illustration of the total, direct, and indirect (mediation) statistical models run in the study.

As one of our main objectives was to test whether HP subfield VR drove PHS’s influence on memory, we further conducted a mediation analysis to test whether HP subfield VR was a potential mediator between PHS (independent variable) and memory score (outcome variable). A statistic toolbox (PROCESS Procedure for SPSS Release 2.16.3),^3^ a validated, freely available computational tool, was used. First, we tested whether the primary independent variable, in this case PHS, predicted the outcome measure, memory score. Next, we tested the direct effects of the primary predictor on the mediator and the direct relationship between the mediator and the outcome. Finally, we tested the indirect mediating effect or the extent to which the relationship between PHS and memory score operates statistically through HP subfield VR. We set the Bootstrap samples = 5,000, 95% confidence level for confidence intervals and we control age, gender, and education on the mediator and outcome. Figure 2 illustrates the direct and indirect (mediation) statistical models graphically. The outcome of the indirect effect was considered statistically significant (*p* < 0.05, two-tailed) when zero is not included in the 95% confidence interval (Preacher and Hayes, 2004; Hayes, 2013).

## Results

### Characteristics of the study population

Table 1 shows the demographic and clinical characteristics of our cohort. Three groups did not differ in terms of age (*p* = 0.474) and gender (*p* = 0.460). However, the NC group had a slightly higher educational level than the AD group (*p* = 0.015). There were significant differences in MMSE, CDR, PHS, and harmonized memory composite scores among the three groups.

**TABLE 1:** Demographic and clinical characteristics of NC, MCI, and AD.

Variable	NC *N* = 51	MCI *N* = 69	AD *N* = 43	*p*-value
	Mean (SD)	Mean (SD)	Mean (SD)	
Age	73.71 ± 5.88	73.32 ± 7.40	75.00 ± 8.13	0.474
Education	16.49 ± 2.77^ac^	16.06 ± 2.80	15.05 ± 2.98^ac^	0.046
Gender (F/M)	23/28	28/41	14/29	0.460
APOE4 (±)	15/36	38/31	33/10	<0.001
MMSE	29.06 ± 1.42^ab^, ^ac^	27.65 ± 1.60^ab,bc^	22.63 ± 2.64^ac^, ^bc^	<0.001
CDR	0^ab,ac^	0.5^ab^, ^bc^	0.78 ± 0.27^ac^, ^bc^	<0.001
PHS	0.026 ± 0.607^ab^, ^ac^	0.497 ± 0.825^ab^	0.778 ± 0.808^ac^	<0.001
Memory	0.814 ± 0.498^ab^, ^ac^	0.101 ± 0.417^ab,bc^	−0.699 ± 0.386^ac,bc^	<0.001

^a^NC.

^b^MCI.

^c^AD.

^ab^significant difference *p* < 0.05 between NC and MCI.

^ac^significant difference *p* < 0.05 between NC and AD.

^bc^significant difference *p* < 0.05 between MCI and AD. Superscripts indicate that the pairwise groups have statistical significance using the LSD (if homogeneity of variance) or Game-Howell (if heterogeneity of variance).

Chi-square test for gender distribution differences assessment.

NC, normal cognition; MCI, mild cognitive impairment; AD, Alzheimer’s disease; SD, standard deviation; MMSE, Mini-Mental State Examination; CDR, Clinical Dementia Rating; PHS, polygenic hazard score; Memory, harmonized composite memory score.

### Effect of disease status and PHS status on hippocampus subfield to entire ipsilateral hippocampus volume ratio

Among the total participants’ pool, the PHS status was partitioned into two groups, with either high (∼84‰) or low PHS (∼16‰). This point was defined by Tan et al. (2018) using ADNI data. The interaction effect of disease status (NC, MCI, and AD) and PHS status (high and low PHS) on the right fimbria-to-hippocampus volume ratio (R. fimbria/hippocampus VR) was statistically significant (see Table 2). The main effect of PHS was not statistically significant on R. fimbria/hippocampus VR, and the main effect of diagnosis was statistically significant on it as shown in Table 2. There was no significant interaction effect by PHS status × diagnosis found on other hippocampus subfields to eiHP volume ratio (*p* > 0.05). In the *post hoc* analysis, AD had significantly lower R. fimbria/hippocampus VR than NC (*p* < 0.001) and MCI (*p* = 0.013). But there was no significant difference between NC and MCI (*p* = 0.130). Since the only significant interaction effect was observed in the right fimbria, the following analysis was only carried out in the right fimbria.

**TABLE 2:** Effect of diagnosis and PHS status on R. fimbria/hippocampus VR.

	R. fimbria/hippocampus VR		
	SE	F	*p*-value
gender	<0.001	1.817	0.180
age	0.001	11.923	0.001
education	<0.001	0.187	0.666
PHS status	<0.001	<0.001	0.989
diagnosis	<0.001	3.275	0.040
PHS status × diagnosis	<0.001	3.958	0.021*

*p < 0.05 (general linear mixed model controlling age, gender, and education: ΔF = 5.142, *p* < 0.001, ΔR^2^ = 0.211).

R, right; VR, volume ratio; PHS, polygenic hazard score.

### Association of right fimbria/hippocampus volume ratio with memory

To examine the relationships between right fimbria/hippocampus volume ratio and memory score, partial correlation tests were performed in all subjects and within three diagnostic groups adjusted for age, gender, education, and APOE. As expected, a positive correlation between right fimbria/hippocampus VR and memory score was found in all subjects (*r* = 0.293, *p* < 0.001). Correlation between them was observed in NC (*r* = 0.144, *p* = 0.335), MCI (*r* = 0.222, *p* = 0.075), and AD (*r* = 0.385, *p* = 0.016).

### Effect of PHS status on right fimbria/hippocampus volume ratio with memory

**FIGURE 3:**
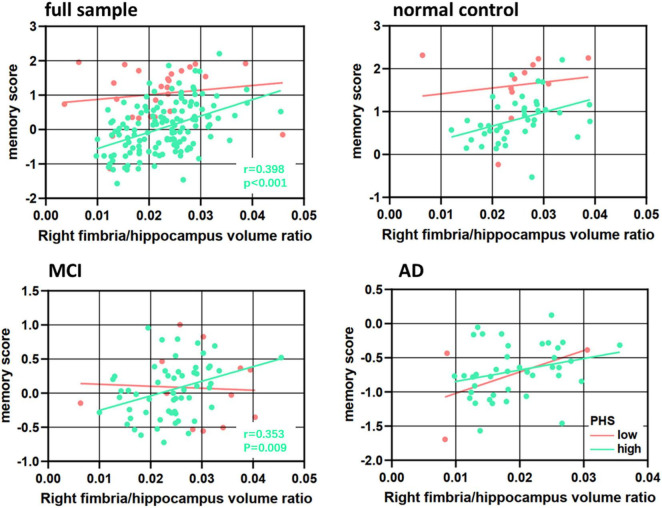
Association between right fimbria/hippocampus volume ratio and memory score in polygenic hazard score (PHS) low and high groups in the full sample, normal controls, mild cognitive impairment (MCI), and Alzheimer’s disease (AD).

In the overall sample, high PHS status had lower memory scores compared to low PHS status (*p* = 0.021) (Table 3). In diagnosis-stratified analyses, high PHS status, compared to low PHS status, had lower memory scores (*p* = 0.020) and larger right fimbria/hippocampus volume ratio (*p* = 0.002) in the MCI, but not in either the NC or AD. Figure 3 shows that the high genetic risk for ADs outperform low-risk group on memory score among subjects with a large right fimbria/hippocampus volume ratio (right side of the x-axis) but this advantage gradually disappears and reverses to confer memory deficits among subjects with moderate to small VR (left side of the x-axis). Among full sample and MCIs with high PHS status, a lower volume ratio was associated with lower memory scores (full sample high PHS group: *p* < 0.001, *r* = 0.398; MCI high PHS group: *p* = 0.009, *r* = 0.353; controlling age, gender and education, Figure 3).

**TABLE 3:** Memory score and right fimbria/hippocampus volume ratio by diagnostic groups and PHS status.

	High PHS	Low PHS	*p*-value
**Full sample**			
Memory score	0.056 ± 0.723	0.411 ± 0.631	0.021*
R. fimbria/hippocampus VR	0.023 ± 0.007	0.022 ± 0.009	0.392
**NC**			
Memory score	0.817 ± 0.531	0.804 ± 0.376	0.944
R. fimbria/hippocampus VR	0.025 ± 0.006	0.025 ± 0.008	0.851
**MCI**			
Memory score	0.047 ± 0.399	0.354 ± 0.423	0.020*
R. fimbria/hippocampus VR	0.024 ± 0.006	0.018 ± 0.006	0.002*
**AD**			
Memory score	–0.691 ± 0.386	–0.803 ± 0.445	0.635
R. fimbria/hippocampus VR	0.019 ± 0.006	0.024 ± 0.019	0.302

*p < 0.05 (Student’s t test in all subjects and within diagnostic groups).

R, right; VR, volume ratio; NC, normal control; MCI, mild cognitive impairment; AD, Alzheimer’s disease; PHS, polygenic hazard score.

### PHS moderates the relationship between hippocampal subfield-to-eiHP volume ratio and memory

Polygenic hazard score moderated the relationship between the right fimbria volume ratio and memory, such that patients with high PHS and lower volume ratio had lower episodic memory composite scores (ΔF = 6.730, *p* = 0.011, Δ*R*^2^ = 0.059) (Table 4, Model 3). To parse the interaction effect, partial correlations were used to examine the relationship between right fimbria volume ratio and memory for low and high PHS. Adjusting for all covariates, results revealed that among patients with high PHS, a lower volume ratio was associated with lower memory score (high: *r* = 0.398, *p* < 0.001, Figure 3). No significant correlation was found in the low PHS group (low: *r* = −0.076, *p* = 0.731, Figure 3). Results revealed a significant difference (Fisher’s z statistic: *z* = −3.2698, *p* = 0.0011), indicating a stronger association between right fimbria/hippocampus volume ratio and memory among patients with high PHS compared to those with low PHS.

**TABLE 4:** Regression analysis for association with right fimbria/hippocampus volume ratio in MCI and AD groups.

	Model 1				Model 2				Model 3			
Variable	B	SE (B)	β	*p*	B	SE(B)	β	*p*	B	SE(B)	β	*p*
Gender	0.118	0.111	0.102	0.288	0.060	0.107	0.052	0.575	0.066	0.105	0.057	0.531
Age	−0.006	0.007	−0.087	0.349	−0.005	0.007	−0.068	0.487	−0.005	0.007	−0.071	0.455
Eduation	0.053	0.019	0.274	0.005	0.046	0.018	0.239	0.011	0.045	0.017	0.233	0.010
PHS					−0.128	0.064	−0.188	0.048	−0.561	0.178	−0.824	0.002
R.fim Volume ratio					21.847	7.286	0.277	0.003	9.321	8.584	0.118	0.280
PHS × R.fim Volume ratio									20.159	7.771	0.707	0.011
*R* ^2^	0.056				0.141				0.185			
Model F	3.204				4.641				5.198			

PHS, polygenic hazard score; R. fim, right fimbria.

### Association between PHS and hippocampal subfield-to-eiHP volume ratio along the Alzheimer’s disease continuum

Diagnosis-stratified analyses were conducted for the right fimbria/hippocampus volume ratio which had a significant PHS × volume ratio interaction on memory. There was a significant interaction between right fimbria/hippocampus volume ratio and PHS in MCI (ΔF = 4.519, *p* = 0.038, Δ*R*^2^ = 0.050) (Table 5, Model 3). This relationship was only significant for MCIs with high PHS (*r* = 0.353, *p* = 0.009, Figure 3C). More specifically, the association between right fimbria/hippocampus volume ratio and memory was stronger in the high PHS group (*r* = 0.353, *p* = 0.009) compared to the low PHS group (*r* = −0.327, *p* = 0.391) in MCI. A significant main effect of PHS (*r* = −0.579, *p* = 0.003) in the MCI group was observed, such that higher genetic risk was associated with lower memory scores. No interactions were present in NC or AD.

**TABLE 5:** Regression analysis for association with right fimbria/hippocampus volume ratio in MCI groups.

	Model 1				Model 2				Model 3			
Variable	B	SE(B)	β	p	B	SE(B)	β	p	B	SE(B)	β	p
Gender	0.095	0.106	0.113	0.372	0.034	0.100	0.041	0.734	0.053	0.098	0.063	0.588
Age	0.000	0.007	0.003	0.981	−0.004	0.007	−0.064	0.588	−0.005	0.006	−0.090	0.437
Eduation	0.055	0.018	0.367	0.004	0.054	0.017	0.365	0.002	0.049	0.017	0.328	0.005
PHS					−0.198	0.062	−0.391	0.002	−0.579	0.189	−1.144	0.003
R.fim Volume ratio					16.884	7.248	0.272	0.023	6.161	8.672	0.099	0.480
PHS × R.fim Volume ratio									16.672	7.842	0.857	0.038
R^2^	0.079				0.203				0.245			
Model F	2.950				4.459				4.676			

PHS, polygenic hazard score; R. fim, right fimbria.

### Mediation analysis of PHS on memory performance

**FIGURE 4:**
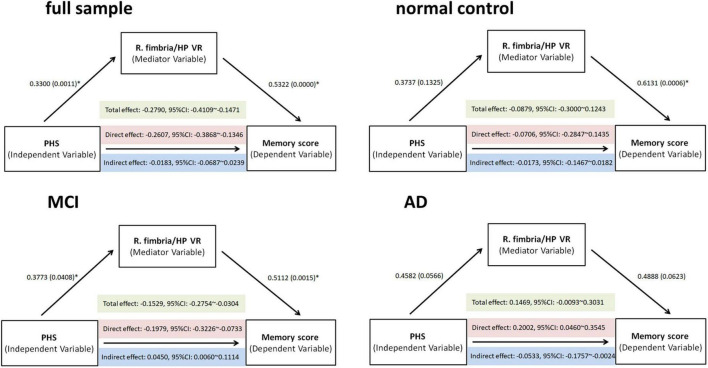
Total, direct, and indirect (mediated) effects of PHS on memory scores were estimated through regression modeling in the full sample, normal controls, mild cognitive impairment (MCI), and Alzheimer’s disease (AD). The effect through the mediating variable—R. fimbria/hippocampus VR—was significant (if bootstrapping 95% CI did not include zero). PHS: polygenic hazard score, R, right, VR, volume ratio; CI, confidence interval.

Mediation models were performed to test the hypothesis that HP subfield VR contributed to PHS-related memory effects. So, we tested the right fimbria/hippocampus volume ratio in all subjects and within three diagnostic groups (Figure 4).

Mediation analyses revealed a partial mediating effect in the MCI group: (i) a significant total effect of PHS value on the memory score (effect size = −0.1529, *p* = 0.0015); (ii) a significant direct effect of PHS value on the memory score (effect size = −0.1979, *p* = 0.0023); (iii) significant indirect effects of increased PHS value on the better memory score mediated by VR (effect size = 0.0450, bootstrapping: standard error = 0.0265, 95%CI: 0.0060–0.1114) (Figure 4). Therefore, PHS might affect memory performance through a change in the R. fimbria/hippocampus VR. No significant mediation effect in the full sample, NC, or AD group was observed.

### All of the analyses include APOE genotype as a covariate

#### Characteristics of the study population

We have added the APOE state in Table 1.

#### Effect of disease status and PHS status on hippocampus subfield to entire ipsilateral hippocampus volume ratio

The interaction effect of disease status (NC, MCI, and AD) and PHS status (high and low PHS) on the right fimbria-to-hippocampus volume ratio (R. fimbria/hippocampus VR) was statistically significant (Supplementary Table 6: *p* = 0.020, general linear mixed model controlling age, gender, education, and APOE: Δ*F* = 4.742, *p* < 0.001, Δ*R*^2^ = 0.172). There was no significant interaction effect by PHS status × diagnosis found on other hippocampus subfields to eiHP volume ratio (p > 0.05).

#### Association of right fimbria/hippocampus volume ratio with memory

To examine the relationships between right fimbria/hippocampus volume ratio and memory score, partial correlation tests were performed in all subjects and within three diagnostic groups adjusted for age, gender, education, and APOE. As expected, a positive correlation between right fimbria/hippocampus VR and memory score was found in all subjects (*r* = 0.293, *p* < 0.001). Correlation between them was observed in NC (*r* = 0.144, *p* = 0.335), MCI (*r* = 0.222, *p* = 0.075), and AD (*r* = 0.385, *p* = 0.016).

#### Effect of PHS status on right fimbria/hippocampus volume ratio with memory

Among full sample and MCIs with high PHS status, a lower volume ratio was associated with lower memory scores (full sample high PHS group: *p* < 0.001, *r* = 0.364; MCI high PHS group: *p* = 0.008, *r* = 0.360; controlling age, gender, education, and APOE).

#### PHS moderates the relationship between hippocampal subfield-to-eiHP volume ratio and memory

Polygenic hazard score moderated the relationship between right fimbria volume ratio and memory, such that patients with high PHS and lower volume ratio had lower episodic memory composite scores (Δ*F* = 4.730, *p* = 0.010, Δ*R*^2^ = 0.190) (Supplementary Table 7, Model 3). To parse the interaction effect, partial correlations were used to examine the relationship between right fimbria volume ratio and memory for low and high PHS. Adjusting for all covariates including APOE, results revealed that among patients with high PHS, lower volume ratio was associated with lower memory score (high: *r* = 0.428, *p* < 0.001). No significant correlation was found in the low PHS group (low: *r* = −0.389, *p* = 0.212).

#### Association between PHS and hippocampal subfield-to-eiHP volume ratio along the Alzheimer’s disease continuum

Diagnosis-stratified analyses were conducted for the right fimbria/hippocampus volume ratio which had a significant PHS × volume ratio interaction on memory. There was a significant interaction between right fimbria/hippocampus volume ratio and PHS in MCI (ΔF = 3.945, *p* = 0.039, Δ*R*^2^ = 0.233) (Supplementary Table 8, Model 3). No interactions were present in NC or AD.

#### Mediation analysis of PHS on memory performance

Mediation analyses revealed a suppression effect in the MCI group: (i) a non-significant total effect of PHS value on the memory score (effect size = −0.1153, *p* = 0.3334); (ii) a non-significant direct effect of PHS value on the memory score (effect size = −0.2036, *p* = 0.0973); and (iii) significant indirect effects of increased PHS value on the better memory score mediated by VR (effect size = 0.0883, bootstrapping: standard error = 0.0495, 95%CI: 0.0127–0.2129) (Supplementary Figure 2). Therefore, PHS without APOE4 might affect memory performance through a change in the R. fimbria/hippocampus VR. But memory performance in MCI can be indicated by a multitude of imaging cues rather than one specific pattern through the right fimbria/hippocampus volume ratio change. No significant mediation effect in the full sample, NC or AD group was observed.

### Executive function analysis for validation

#### Association of right fimbria/hippocampus volume ratio with executive function

To examine the relationships between right fimbria/hippocampus volume ratio and executive function score, partial correlation tests were performed in all subjects and within three diagnostic groups adjusted for age, gender, education, and APOE. As expected, a positive correlation between right fimbria/hippocampus VR and executive function score was found in all subjects (*r* = 0.181, *p* = 0.023). Correlation between them was observed in and NC (*r* = 0.361, *p* = 0.013), MCI (*r* = 0.046, *p* = 0.717), and AD (*r* = 0.011, *p* = 0.949).

#### Effect of PHS status on right fimbria/hippocampus volume ratio with executive function

There was no significant difference found in executive function scores between high and low PHS groups in full sample, NC, MCI, and AD (Supplementary Table 9). Among full sample and NCs with high PHS status, a lower volume ratio was associated with lower executive function scores (full sample high PHS group: *p* = 0.001, *r* = 0.278; NC high PHS group: *p* = 0.004, *r* = 0.464; controlling age, gender, and education).

#### PHS moderates the relationship between hippocampal subfield-to-eiHP volume ratio and executive function

Polygenic hazard score tended to moderate the relationship between the right fimbria volume ratio and executive function, such that patients with high PHS and lower volume ratio had lower executive function composite scores (Δ*F* = 3.053, *p* = 0.051, Δ*R*^2^ = 0.100).

#### Association between PHS and hippocampal subfield-to-eiHP volume ratio along the Alzheimer’s disease continuum

Diagnosis-stratified analyses were conducted for the right fimbria/hippocampus volume ratio which had a significant PHS × volume ratio interaction on executive function. No interactions were present in NC, MCI, or AD.

#### Mediation analysis of PHS on executive function

Mediation models were performed to test the hypothesis that HP subfield VR contributed to PHS-related executive function effects. So, we tested the right fimbria/hippocampus volume ratio in all subjects and within three diagnostic groups (Supplementary Figure 3).

Mediation analyses revealed a direct effect in full sample: (i) a significant total effect of the PHS value on executive function score (effect size = −0.1367, *p* = 0.0345); (ii) a significant direct effect of the PHS value on executive function score (effect size = −0.1259, *p* = 0.0483); and (iii) non-significant indirect effects mediated by VR (effect size = −0.0108, bootstrapping: standard error = 0.0156, 95%CI: −0.0516 to 0.0118) (Supplementary Figure 3). Therefore, PHS might affect executive function performance directly or through other pathways rather than the right fimbria/hippocampus volume ratio. No significant mediation effect in the NC, MCI, or AD group was observed (Supplementary Figure 3).

## Discussion

The purpose of the current study was to examine neurobiological markers that influence cognitive performance related to AD. In this study, we investigated the effect of the PHS and hippocampal subfield volume ratios on a previously validated episodic memory composite score in the three disease status groups (NC, MCI, and AD). There were three main findings. First, a significant interaction between disease status and PHS status was primarily observed in the right fimbria. Second, patients with high PHS and lower right fimbria/hippocampus volume ratio had lower episodic memory composite scores. After conducting diagnosis-specific analyses, this interaction was only observed in the MCI group. Finally, the right fimbria/hippocampus volume ratio partially mediated the effects of PHS on memory performance in the MCI group. Together, these findings suggest that hippocampal subfield volume ratio and polygenic risk for AD represent important markers of episodic memory performance.

We observed no main effect of PHS status but prominent interaction between PHS and disease status (NC, MCI, and AD) in the right fimbria. This finding, although counter-intuitive, is supported by the previous work suggesting that brain injury in AD results from interactions between disease status and PHS (Prieto et al., 2020). We confirmed that the effect of genetic risks for AD on the hippocampus was driven by disease status (e.g., the clinical symptoms). Longitudinal studies exploring healthy aging and transition to MCI and AD will provide further clarity regarding these genetic effects. Further, our volume ratio analysis revealed a decreased VR in the right fimbria in the AD group, compared to the MCI and NC groups. This is reasonable. The fimbria is a white matter structure that extends from the alveus and eventually forms the fornix. It carries axons that emanate primarily from pyramidal neurons in the CA1 and subiculum (Joseph and Cardozo, 2004). In the previous study, a smaller volume of fimbria showed strong associations with poor cognitive/memory performance controlling for total hippocampal volume (Evans et al., 2018) and was commonly found in patients with MCI and AD. This may be, in part, due to its anatomical connections and location within functional pathways. For instance, the fimbria–fornix (FF) bundle, through which noradrenergic and cholinergic afferents reach the hippocampal formation, profoundly affects memory (Buzsáki et al., 1992; Cassel et al., 1997). Thus, the FF-lesion has been used as a model of age-dependent memory deficits (Mercerón-Martínez et al., 2020). So, its integrity is important in preserving the hippocampus’s key role in memory (Nilsson et al., 1987). A recent QSM study showed that the magnetic susceptibility of the fimbria was greater in patients with AD, implying microstructural changes in the tissue (Au et al., 2021). The authors believed that the susceptibility changes may also account for the downstream reduction in hippocampal/fimbrial volume. Diffusion tensor imaging study also has shown changes in the fimbria which were able to explain some variance in the memory tests (Cahn et al., 2021). Despite these promising findings, little is currently known about the biological underpinnings of a fimbria/hippocampus volume ratio, as previous models of neuropathology have primarily focused on the biological mechanisms involved in hippocampal atrophy.

Alterations in the output of hippocampus information due to alterations in the integrity of the fimbria could explain the patients’ reduced memory performance. This proposal seems to be further reinforced by the observed solid and direct correlations between the right fimbria/hippocampus VR and memory score (Figure 3). Our findings are also supported by previous studies, which have shown the reduced fimbria volume and associated altered memory in rats (Winters and Dunnett, 2004; Addy et al., 2005), asymptomatic adults (Tomaiuolo et al., 2004; Zheng et al., 2018), and patients with multiple sclerosis (González Torre et al., 2017). This observation highlights the importance of fine grain analyses at specific hippocampal subfields to detect correlates of memory deficits. In line with this, the destructive effect of the APOEε4 allele on white matter tracts in the right fornix was revealed in old participants, which was positively associated with memory impairment (Gold et al., 2010; Zhang et al., 2015). These findings propose the effects of fimbria volume and genetic risk for AD on memory preceding the development of dementia.

Results of this study showed the utility of the PHS to assess the current levels of hippocampus subfield atrophy and episodic memory function in old people. It is still not fully known through which mechanisms PHS impact AD risk. Still, hippocampus atrophy level and cognitive function are considered intermediate phenotypes that may mediate genetic effects on AD risk (Desikan et al., 2017; Kauppi et al., 2018). Previous studies using both survival analyses and linear mixed effect models showed an improved prediction of AD progression by combining MRI data with cognitive performance, and genetic risk for AD (McEvoy et al., 2009; Li et al., 2016; Kauppi et al., 2018). The predictive capabilities of PHS may have contributed to our finding of genetics moderating the relationship between the right fimbria/hippocampus VR and episodic memory. Similar findings were observed in a prior study in old adults (Prieto et al., 2020) that the PHS moderated episodic memory through the left hippocampus in the MCI group.

Whereas our moderation analysis results are consistent with those previously found for PHS (Prieto et al., 2020), our meditational analyses reported novel findings. Mediation models in cognitive neurogenetic research provide a useful framework for directional hypotheses, which are generally appropriate for cognitive neurogenetics because effects generally transmit from genes to brain and cognition, rather than vice versa. The strong total, direct effects, and partial indirect effect of the PHS on episodic memory through the right fimbria/hippocampus VR may reflect specific hippocampus subfield atrophy patterns in differential diagnosis and prognosis of AD (Mizutani and Kasahara, 1997; de Flores et al., 2015; Zeng et al., 2021), which in turn alters episodic memory performance. The data indicate a gene–brain–cognition effect pathway (Green et al., 2013), whereby PHS influences episodic memory *via* downregulation in the right fimbria volume ratio in the MCI group. Investigators reported genetic variants influencing hippocampal subfield volume also modified the risk of developing AD (Hibar et al., 2017). The larger volume of the hippocampus subfields might have translated into behavioral advantages. Moreover, neuronal activity within specific subfields of the hippocampus might serve distinct aspects of memory (Squire, 2004; Mueller et al., 2011). Therefore, subfield-specific effects of PHS might further underline a proposed role for genotype in particular aspects of memory formation. For example, fimbria appears to support episodic detail generation, as well as retrieval of other types of episodic content (St-Laurent et al., 2014, 2016; Memel et al., 2020). Improved volume and better microstructure in fimbria have been associated with better memory performance in MCI subjects (Pereira et al., 2016; Kantarci et al., 2017; Berron et al., 2020). Thus, the observed decreased volume ratio in fimbria could have contributed to poorer memory results with high PHS status.

Interestingly, the moderate and partial mediated effect was most evident among patients with MCI, indicating that the complicated pathological process related to hippocampus atrophy and memory performance changes along the Alzheimer’s disease continuum. Genetic effects known to contribute to AD pathogenesis can modulate adult hippocampal neurogenesis (Berron et al., 2020). Notably, these alterations may occur at the very early stage of AD progression, prior to processes like neuronal loss and amyloid deposition which might lead to memory impairment (Jin et al., 2004; Mu and Gage, 2011). Further, episodic memory was also affected in the earliest stages of AD (Backman et al., 2001; Gold and Budson, 2008). This is consistent with functional imaging studies showing that in the first few years of AD different hippocampal subfields were affected in memory network organization and maintenance (Li et al., 2009; Yassa et al., 2010; Sun et al., 2017). In the AD group, mediation analyses revealed a suppression effect: we found no significant total effect of PHS on memory (effect size = 0.1469, 95%CI: −0.0093 to 0.3031), while the indirect effect of PHS on memory was negative (effect size = −0.0533, 95%CI: −0.1757 to −0.0024) and the direct effect was positive (effect size = 0.2002, 95%CI: 0.0460–0.3545). It is possible that by the time individuals progress to AD, even the combination of genetic risk and hippocampus subfield volumes can no longer explain the variance in episodic memory performance. Because these patients with AD probably suffer from great and multiple brain regions volume loss in structural plasticity and further along a cognitive decline trajectory. A promising avenue for future research on this topic could be the construct of multiple subfields of atrophy patterns as the mediate variable to test the effects of PHS on memory. Considering APOE is strongly associated with both cognition and hippocampal volumes even in very early cases (Haller et al., 2019; Herrmann et al., 2019), we have completed all the analyses including APOE genotype as a covariate. We found that a PHS score without APOE4 contribution keeps the same effect.

In addition, in our executive function analysis for validation, we found that the right fimbria/hippocampus VR had distinct associations with executive function. PHS tended to moderate the relationship between right fimbria volume ratio and executive function, such that patients with high PHS and lower volume ratio might have lower executive function composite scores. Significant total and direct effect but no significant mediation effect was found to test the hypothesis that HP subfield VR contributed to PHS-related executive function effects. The reason why this result was found in the executive function data may be that executive function performance can be indicated by a multitude of imaging cues rather than one specific pattern through the right fimbria/hippocampus volume ratio change. A promising avenue for future research on this topic could be the construct of multiple subfields of atrophy patterns as the mediate variable to test the effects of PHS on executive function. The results highlighted a specific pathway in which PHS affects memory rather than executive function by degrading the right fimbria/hippocampus VR.

## Limitation

Our study has several limitations. First, we used T1-weighted images provided by a 3T MR scanner for hippocampal subfield segmentation. Sometimes it may be challenging to determine the boundaries, especially in several small volume subfields. Future studies using 7T MRI would be more precise to measure subfields. Second, since this was the first study to explain a potential pathway associating PHS-related episodic memory alteration with the hippocampus subfield volume ratio modification by structural MRI in old adults (all the MCIs were amnestic MCIs), studies in different cohorts, or recruiting a larger and more diverse population, may be needed to extend our findings. Third, because of the cross-sectional and retrospective design, we were unable to prove that alterations in hippocampus subfield volume ratios were actually a consequence of the high genetic risk for AD. Lastly, there must be other variables affecting the relationship between the hippocampus and memory performance that need to be further discussed.

## Conclusion

The present study demonstrated the mediating effect of hippocampal subfield volume ratio and the moderating effect of polygenetic risk for AD between hippocampus subfield volume ratio and episodic memory performance. Genetic risk and hippocampal subfields should be considered as key variables in models tracking the progression of cognitive decline in healthy and pathological aging.

## Data availability statement

The original contributions presented in the study are included in the article/Supplementary material, further inquiries can be directed to the corresponding author.

## Ethics statement

The studies involving human participants were reviewed and approved by the Ethics committee of the Second Affiliated Hospital of Zhejiang University, School of Medicine. The patients/participants provided their written informed consent to participate in this study. Written informed consent was obtained from the individual(s) for the publication of any potentially identifiable images or data included in this article.

## Author contributions

JX designed the study and wrote the first draft of the manuscript. XG analyzed the MRI data. JW, MZ, and XX assisted with the study design and interpretation of findings. All authors have contributed to and approved the final manuscript.

## Footnotes

1
adni.loni.usc.edu

2
http://surfer.nmr.mgh.harvard.edu

3
http://www.afhayes.com/index.html
