# Supplementary material for: Polygenic hazard score modified the relationship between hippocampal subfield atrophy and episodic memory in older adults
Source: Front Aging Neurosci. 2022 Oct 31;14:943702. doi: 10.3389/fnagi.2022.943702 (PMC9659745; doi:10.3389/fnagi.2022.943702)
Supplement: Supplementary file 1 [file Data_Sheet_1.PDF]

Supplementary Material

1 Supplementary Data

1.1 To verify hippocampal subfield-to- eiHP volume ratio (VR) measurement method

Whilst most in vivo human studies linking hippocampus to AD risk and memory impairment have focused on its gross volume, animal and pathological studies reveal the hippocampus to contain anatomical subregions with corresponding functional specialisation (Mueller et al., 2011; Tamnes et al., 2014; Yassa et al., 2011). Considering that these structures are all part of the hippocampus and are structurally and functionally connected, examining these structures in the context of one another may be more informative than studying each region in isolation.

VR is a measure combining the assessment of subfield volume with whole hippocampal volume. By providing in a single measure both a standard volumetric assessment of the hippocampal subfield and an approximation of volume loss, based on hippocampal atrophy, we believe the VR provides a superior cross-sectional estimation of hippocampal structural integrity.

In order to verify this measurement method, we compared the relationship between VR and standard hippocampal subfield volume and memory performance in old healthy and AD cohorts. In both cohorts, the correlation between VR and memory performance was numerically superior to the standard volume in some hippocampal subfields (Supplementary Table 1). To a certain extent, these findings support the advantage of using VR over standard volume when examining the relationship with memory function among AD. Although further validation is needed, we propose that the calculation of VR is a promising method that can improve the assessment of hippocampal integrity from cross-sectional MR images.

1.2 Hippocampal subfield-to- eiHP volume ratio (VR) measurement in three groups

Both MCI and AD patients showed decreased VR in left ML, and increased VR in right HP fissure than NC ($p < 0.05$, Bonferroni corrected) controlling for age, education, and gender (Supplementary Table 2). Also, AD patients showed more VR than NC in the left HP fissure, right CA1 and right CA4, showed less VR in right subiculum and right fimbria (Supplementary Table 2).

1.3 Mediation analyses for the effect of PHS on memory score mediated by R. fimbria/hippocampus VR, while controlling for age, gender and education in MCI group

Mediation analyses revealed a partial mediating effect in MCI group: (i) a significant total effect of PHS value on memory score (effect size =-0.1529, P = 0.0015); (ii) a significant direct effect of PHS value on memory score (effect size =-0.1979, P = 0.0023); (iii) significant indirect effects of increased PHS value on better memory score mediated by VR (effect size = 0.0450, bootstrapping: standard error = 0.0265, 95%CI: 0.0060 to 0.1114) (Supplementary Figure 1, Supplementary Table 3).

1.4 To validate that PHS moderates the relationship between hippocampal subfield-to-eiHP volume ratio and memory

We have included a model with the interaction alone (Table S4 and S5). The results are stable: PHS moderated the relationship between right fimbria volume ratio and memory, such that patients with high PHS and lower volume ratio had lower episodic memory composite scores [ΔF =7.520, p = 0.011, ΔR2 = 0.150] (Supplementary Table 4 Model 3). There was a significant interaction between right fimbria/hippocampus volume ratio and PHS in MCI [ΔF= 5.739, p = 0.015, ΔR2 = 0.173] (Supplementary Table 5 Model 3).

2 Supplementary Figures and Tables

Supplementary Table 1 the correlation between VR or Standard volume and memory performance

	Old healthy control				AD			
	VR		Standard volume		VR		Standard volume	
	p	r	p	r	p	r	p	r
L.HP tail	0.616	0.074	0.545	0.091	0.949	0.011	0.663	0.072
L. subiculum	0.167	-0.203	0.742	-0.049	0.712	0.061	0.357	0.151
L.CA1	0.391	-0.127	0.841	-0.030	0.064	-0.300	0.552	-0.098
L.HP fissure	0.003	-0.416	0.021	-0.337	0.122	-0.251	0.250	-0.189
L.presubiculum	0.643	0.069	0.518	0.097	0.331	0.160	0.234	0.195
L.parasubiculum	0.078	0.257	0.093	0.248	0.871	-0.027	0.850	0.031
L.ML	0.753	-0.047	0.786	0.041	0.581	0.091	0.379	0.145
L.GCMLDG	0.944	0.010	0.651	0.068	0.786	-0.045	0.582	0.091
L.CA3	0.695	0.058	0.585	0.082	0.919	-0.017	0.670	0.070
L.CA4	0.653	-0.067	0.845	0.029	0.656	-0.074	0.647	0.076
L.fimbria	0.764	0.044	0.546	0.090	0.241	0.192	0.213	0.204
L.HATA	0.827	-0.032	0.892	0.020	0.248	0.190	0.191	0.214
R. HP tail	0.637	0.070	0.831	-0.032	0.612	-0.084	0.468	-0.120
R.subiculum	0.385	-0.128	0.201	-0.190	0.823	-0.037	0.512	-0.108
R.CA1	0.507	-0.098	0.259	-0.168	0.723	-0.059	0.346	-0.155
R. HP fissure	0.007	-0.385	0.005	-0.401	0.218	-0.202	0.144	-0.238
R.presubiculum	0.248	-0.170	0.149	-0.214	0.756	0.051	0.640	-0.077
R.parasubiculum	0.781	-0.041	0.489	-0.103	0.145	0.238	0.343	-0.156
R.ML	0.449	-0.112	0.375	-0.132	0.102	0.266	0.704	-0.063
R. GCMLDG	0.471	0.107	0.670	0.064	0.646	-0.076	0.400	-0.139
R.CA3	0.678	0.062	0.873	-0.081	0.565	-0.095	0.423	-0.132
R.CA4	0.854	-0.027	0.588	-0.081	0.368	-0.148	0.268	-0.182
R.fimbria	0.229	0.177	0.414	0.122	0.016	0.385	0.053	-0.312
R.HATA	0.142	0.215	0.539	0.092	0.476	-0.117	0.293	-0.173

Abbreviation: L: Left; R: Right; VR: Volume ratio; AD, Alzheimer’s disease; HP, hippocampus; ML, the molecular layer; CA, cornus ammonis; GC-ML-DG, the molecular and granule cell layers of the dentate gyrus; HATA, hippocampal amygdalar transition area.

Supplementary Table 2 Hippocampal Subfield-to-epHippocampus (10^{-2}) of Three Groups

Subfield	NC	MCI	AD	p
Left Tail	14.420±1.634	14.866±1.587	15.003±1.861	0.198
Left Subiculum	12.895±0.765	12.601±0.919	12.426±0.865	0.028
Left CA1	18.945±1.159	19.186±1.376	19.407±1.296	0.225
Left HP fissure	5.457±0.871[ac]	5.870±1.096	6.358±1.129[ac]	<0.001
Left Presubiculum	8.929±0.696	8.884±0.873	8.760±0.984	0.616
Left Parasubiculum	1.950±0.432	1.938±0.424	2.051±0.519	0.403
Left ML	16.595±0.321[ab, ac]	16.371±0.402[ab]	16.209±0.454[ac]	<0.001
Left GC-ML-DG	8.518±0.486	8.538±0.530	8.564±0.487	0.909
Left CA3	6.099±0.621	6.138±0.687	6.184±0.613	0.822
Left CA4	7.364±0.422	7.423±0.478	7.480±0.469	0.475
Left Fimbria	2.494±0.677	2.192±0.776	2.103±0.913	0.037
Left HATA	1.791±0.222	1.865±0.251	1.813±0.278	0.247
Right Tail	14.592±1.418	14.624±1.848	14.808±1.824	0.808
Right Subiculum	12.379±0.605[ac]	12.079±0.792	11.872±0.816[ac]	0.005
Right CA1	19.301±1.009[ac]	19.576±1.058	20.018±1.311[ac]	0.009
Right HP fissure	5.806±0.987[ac, ac]	6.198±1.041[bc]	6.712±0.971[ac]	<0.001
Right Presubiculum	8.086±0.643	7.903±0.699	7.692±0.697	0.022
Right Parasubiculum	1.834±0.389	1.785±0.367	1.736±0.403	0.468
Right ML	16.518±0.389	16.458±0.456	16.307±0.409	0.05
Right GC-ML-DG	8.730±0.481	8.915±0.576	9.023±0.610	0.036
Right CA3	6.590±0.630	6.700±0.630	6.817±0.784	0.269
Right CA4	7.570±0.417[ac]	7.720±0.482	7.917±0.542[ac]	0.003
Right Fimbria	2.486±0.664[ac]	2.292±0.673	1.959±0.736[ac]	0.001
Right HATA	1.914±0.221	1.946±0.228	1.853±0.288	0.144

Abbreviation: NC, normal cognition; MCI, mild cognitive impairment; AD, Alzheimer's disease; HP, hippocampus; ML, the molecular layer; CA, cornus ammonis; GC-ML-DG, the molecular and granule cell layers of the dentate gyrus; HATA, hippocampal amygdalar transition area.

[a]NC,

[b]MCI,

[c]AD,

[ab]significant difference $p<0.05/24=0.002$ between NC and MCI,

[ac]significant difference $p<0.05/24=0.002$ between NC and AD,

[bc]significant difference $p<0.05/24=0.002$ between MCI and AD.

Superscripts indicate that the pairwise groups have statistical significance using the LSD.

Supplementary Table 3 Mediation analyses for the effect of PHS on memory score mediated by R. fimbria/hippocampus VR, while controlling for age, gender and education in MCI group

	Effect	SE	t	p
Model 1 Dependent variable: R. fimbria/hippocampus VR				
Model (r=0.3773, p=0.0408)				
Constant*	0.0275	0.0101	2.7076	0.0087
PHS*	0.0027	0.0010	2.6049	0.0114
Age	-0.0001	0.0001	-0.9790	0.3313
Gender	0.0014	0.0017	0.8126	0.4194
Education	<0.0001	0.0003	0.0683	0.9458
Model 2 Dependent variable: memory score				
Model (r=0.5112, p=0.0015)				
Constant	-0.8431	0.6209	-1.3579	0.1793
R. fimbria/hippocampus VR*	16.8835	7.2482	2.3293	0.0231
PHS*	-0.1979	0.0624	-3.1727	0.0023
Age	-0.0036	0.0066	-0.5444	0.5881
Gender	0.0341	0.1000	0.3416	0.7338
Education*	0.0543	0.0171	3.1718	0.0023

Note: The indirect effect of PHS on memory mediated by right fimbria/hippocampus volume ratio (indirect effect in Figure 2) is assessed with Models 1 and 2. Model 1 determines the impact of PHS on the right fimbria/hippocampus volume ratio, and Model 2 determines the impact of the right fimbria/hippocampus volume ratio on memory score. The direct effect of PHS on memory score (direct effect in Figure 2) is assessed with Model 2.*Variable is a significant predictor at the 0.05 level.

Abbreviation: PHS: polygenic hazard score, R: Right, VR: volume ratio.

Supplementary Table 4. Regression analysis for association with Right fimbria/hippocampus volume ratio in MCI and AD groups

	Model 1				Model 2				Model 3			
Variable	B	SE(B)	β	p	B	SE(B)	β	p	B	SE(B)	β	p
PHS	-0.101	0.064	-0.149	0.118	-0.124	0.061	-0.183	0.045	-0.566	0.180	-0.832	0.002
R.fim Volume ratio					24.921	7.115	0.316	0.001	12.181	8.492	0.155	0.154
PHS× R.fim Volume ratio									20.606	7.930	0.723	0.011
R^2	0.013				0.105				0.150			
Model F	2.489				7.507				7.520			

Abbreviation: PHS: polygenic hazard score, R.fim: Right fimbria.

Supplementary Table 5. Regression analysis for association with Right fimbria/hippocampus volume ratio in MCI groups

	Model 1				Model 2				Model 3			
Variable	B	SE(B)	β	p	B	SE(B)	β	p	B	SE(B)	β	p
PHS	-0.131	0.060	-0.259	0.031	-0.180	0.062	-0.355	0.005	-0.637	0.192	-1.259	0.001
R.fim Volume ratio					16.974	7.584	0.274	0.029	4.535	8.825	0.073	0.609
PHS× R.fim Volume ratio									20.025	7.988	1.030	0.015
R^2	0.053				0.107				0.173			
Model F	4.826				5.062				5.739			

Abbreviation: PHS: polygenic hazard score, R.fim: Right fimbria.

Supplementary Table 6. Effect of diagnosis and PHS status on R. fimbria/hippocampus VR

	R. fimbria/hippocampus VR		
	SE	F	P
gender	<0.001	1.727	0.191
age	0.001	13.318	<0.001
education	<0.001	0.103	0.749
APOE 4	<0.001	1.424	0.235
PHS status	<0.001	0.222	0.638
diagnosis	<0.001	2.872	0.060
PHS status × diagnosis	<0.001	4.031	0.020*

Note: *p<0.05 (General linear mixed model controlling age, gender, education and Aβ: $\Delta F= 4.742$, $p <0.001$, $\Delta R^2 = 0.172$).

Abbreviation: R: Right, VR: volume ratio, PHS: Polygenic Hazard Score, APOE 4: APOE 4 status.

Supplementary Table 7. Regression analysis for association with Right fimbria/hippocampus volume ratio in MCI and AD groups

	Model 1				Model 2				Model 3			
Variable	B	SE(B)	β	p	B	SE(B)	β	p	B	SE(B)	β	p
Gender	0.084	0.109	0.073	0.444	0.063	0.107	0.054	0.558	0.068	0.104	0.059	0.513
Age	-0.011	0.007	-0.155	0.105	-0.006	0.007	-0.078	0.240	-0.006	0.007	-0.082	0.390
Eduation	0.050	0.018	0.259	0.007	0.047	0.018	0.240	0.010	0.045	0.017	0.234	0.010
APOE4	-0.264	0.111	-0.227	0.019	-0.225	0.182	-0.193	0.221	-0.232	0.177	-0.200	0.194
PHS					-0.021	0.108	-0.030	0.850	-0.453	0.196	-0.666	0.023
R.fim Volume ratio					20.919	7.308	0.266	0.005	8.259	8.593	0.105	0.339
PHS× R.fim Volume ratio									20.323	7.745	0.713	0.010
R^2	0.095				0.145				0.190			
Model F	3.929				4.139				4.730			

Abbreviation: PHS: polygenic hazard score, R.fim: Right fimbria, APOE4: APOE4 status.

Supplementary Table 8. Regression analysis for association with Right fimbria/hippocampus volume ratio in MCI groups

	Model 1				Model 2				Model 3			
Variable	B	SE(B)	β	p	B	SE(B)	β	p	B	SE(B)	β	p
Gender	0.053	0.104	0.063	0.610	0.035	0.101	0.041	0.733	0.054	0.099	0.064	0.588
Age	-0.004	0.007	-0.074	0.537	-0.004	0.007	-0.064	0.591	-0.005	0.007	-0.090	0.441
Eduation	0.052	0.018	0.347	0.005	0.054	0.017	0.366	0.003	0.049	0.017	0.329	0.006
APOE4	-0.231	0.100	-0.277	0.024	0.010	0.189	0.012	0.956	0.014	0.184	0.017	0.940
PHS					-0.204	0.121	-0.403	0.097	-0.586	0.216	-1.159	0.009
R.fim Volume ratio					16.961	7.442	0.274	0.026	6.262	8.843	0.101	0.482
PHS× R.fim Volume ratio									16.677	7.906	0.858	0.039
R^2	0.137				0.190				0.233			
Model F	3.691				3.657				3.945			

Abbreviation: PHS: polygenic hazard score, R.fim: Right fimbria, APOE4: APOE4 status.

Supplementary Table 9. Executive function by diagnostic groups and PHS status.

	High PHS	Low PHS	p value
**Full sample**			
Executive function score	0.248±0.669	0.506±0.668	0.074
**NC**			
Executive function score	0.663±0.467	0.918±0.436	0.111
**MCI**			
Executive function score	0.348±0.531	0.452±0.372	0.521
**AD**			
Executive function score	-0.309±0.657	-0.789±0.638	0.229

Note: *p<0.05 (student's t test in all subjects and within diagnostic groups)

Abbreviation: R: Right, VR: volume ratio, NC: Normal control, MCI: mild cognitive impairment, AD: Alzheimer disease, PHS: Polygenic Hazard Score.

3. Supplementary Figures

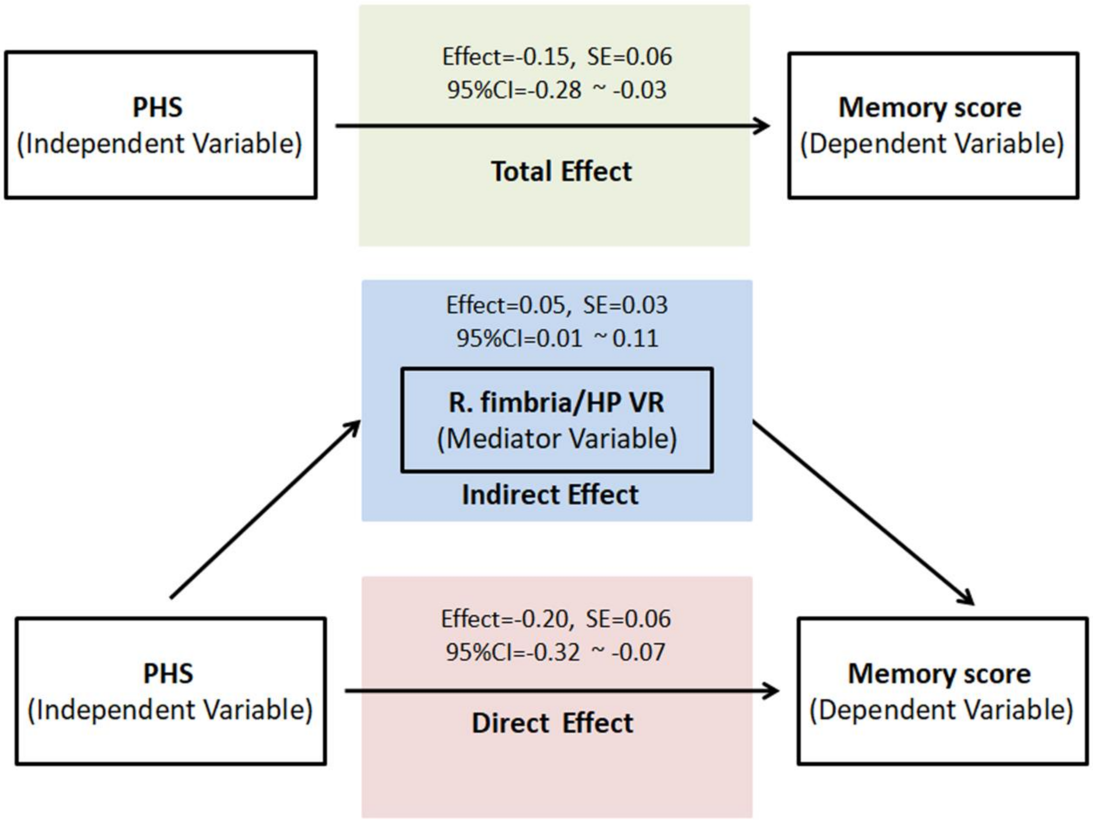

Supplementary Figure 1. Total, direct and indirect (mediated) effects of PHS on memory score estimated through regression modeling in MCI. The direct effect and indirect effect through the

mediating variable—R. fimbria/hippocampus VR—was significant (bootstrapping 95% CI did not include zero). PHS: polygenic hazard score, R: Right, VR: volume ratio, CI: confidence interval.

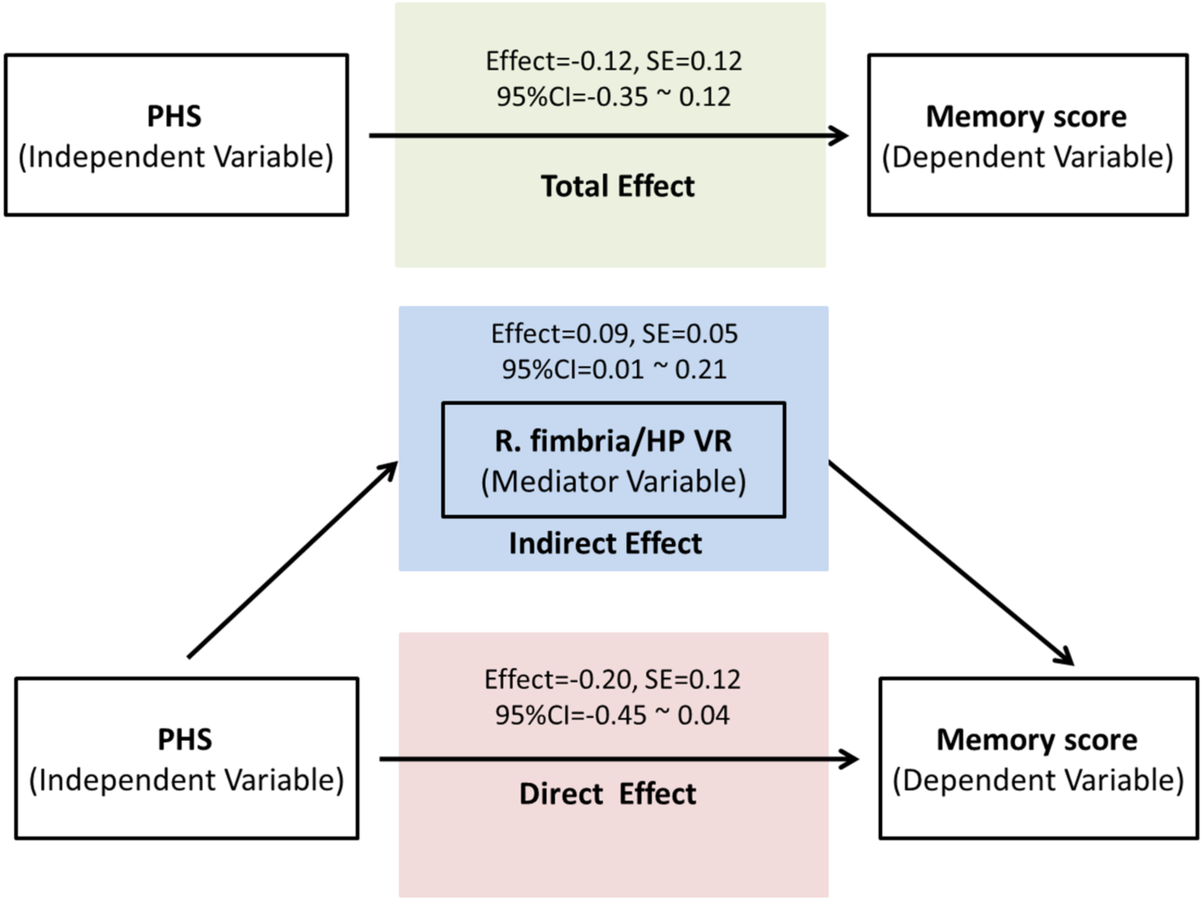

Supplementary Figure 2. Total, direct and indirect (mediated) effects of PHS on memory score estimated through regression modeling in MCI (Covariates included in the model were: gender, age, education and APOE4). The indirect effect through the mediating variable—R. fimbria/hippocampus VR—was significant (bootstrapping 95% CI did not include zero). PHS: polygenic hazard score, R: Right, VR: volume ratio, CI: confidence interval.

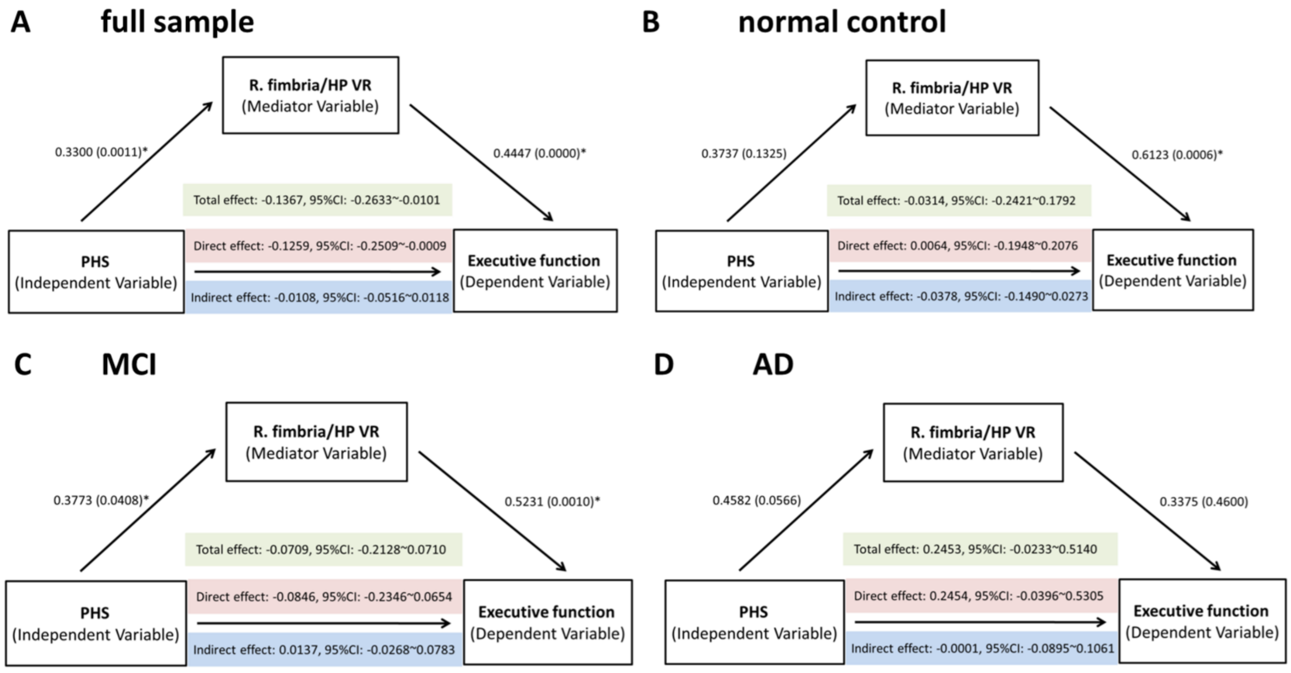

Supplementary Figure 3. Total, direct and indirect (mediated) effects of PHS on Executive function score estimated through regression modeling in the full sample, normal controls, mild cognitive impairment (MCI), and Alzheimer disease (AD). The effect through the mediating variable—R. fimbria/hippocampus VR—was not significant (if bootstrapping 95% CI did not include zero). PHS: polygenic hazard score, R: Right, VR: volume ratio, CI: confidence interval
